# Prize Essay

**Published:** 1886-06

**Authors:** 


### EDITORIAL NOTES.

### PRIZE ESSAY—A SURPRISE IN STORE FOR THE PROFESSION.

The committee appointed by the Texas State Medical Association to award a prize of $100 in gold for the best original essay by any member, received nine papers. They were all long, some longer, and one longest. There is an old saying that "the longest pole knocks the persimmons." In this contest the longest paper, (and the committee thought the best) knocked the prize; it being awarded to Dr. J. R. Briggs, of Ft. Worth, Texas. His paper filled sixty-seven pages of closely type-written follscap paper; and was bound in book form. The subject was:

"*Reflections on Physical and Mental Culture, in reference to Hereditary Predispositions, Action and Reaction of Mind and Body, Habits, Normal Automatic Mind Action, Automatic Mental Action Resulting from Stimulants and Narcotics, Clinical Aspects and Suggestions for Physiological and Psycological Advancement!!!*"

The title is quite a long pole, itself, and if any modern Shakespare would know "what's in a name," just let him take a peep at the prize essay of T. S. M. A.; there is very considerable—length, in it, at least. The "prize essay" will appear in the Transactions, and we predict will be a stunning surprise to the profession of America. The names of the committeemen making the award, are Dr. J. D. Osborn, Cleburne, Chairman; Dr. R. D. Wallace, Terrell; Drs. H. K. Leake, S. D. Thruston and E. L. Thompson, Dallas. Those gentlemen are entitled to the sympathy of the profession, after having read these nine papers, all of which possessed one feature in common, at least,—length.

